# Le Dieu des pauvres: socio-anthropologie de la résistance des villes Camerounaises à la COVID-19

**DOI:** 10.11604/pamj.2021.38.348.27333

**Published:** 2021-04-12

**Authors:** Amada Talikoa, Rose-Danielle Ngoumou

**Affiliations:** 1 Université de Yaoundé 1, Département de Sociologie, Yaoundé, Cameroun

**Keywords:** Socio-anthropologie, COVID-19, résistances, villes camerounaises, Socio-anthropology, COVID-19, resistances, Cameroon cities

## Abstract

**Introduction:**

la COVID-19, depuis son apparition a épuisé les systèmes sanitaires mondiaux. Il était prévisible que les pays avec un système sanitaire peu solide se verront gravement anéantis par la pandémie. Les pays d´Europe ont connu des pertes humaines importantes et on prévoyait que l´Afrique connaitrai un drame encore plus pire. Seulement depuis l´évolution de cette pandémie, on assiste à une remarquable résistance des pays d´Afrique et du Cameroun face à ce virus, comme s´ils vivaient en marge du monde.

**Méthodes:**

l´étude a été phénoménographique. Les données ont été collectées, de façon successive, des observations de médias (notamment les sites de l´OMS, télévisions Crtv (l´émission Parlons COVID), des réseaux sociaux (Facebook et Whatsapp) et des observations directes de quelques quartiers de Garoua (Roumdé-Adjia, Foulbéré, Kakataré) et Mora pour l´Extême-Nord, et la zone Sud de Yaoundé (Ngoa-Ekelé, Nkolondom, Mokolo). Ces observations ont été associées aux entretiens individuels, recensions et prises de notes aux abords des lieux de circulation publique (lieux de culte, marchés et les espaces de causerie; les Faada). Le cadre théorique mobilisé est la théorie du fonctionnalisme.

**Résultats:**

les résultats montrent que les Camerounais perçoivent la pandémie comme un phénomène éminemment métasocial ce qui explique leur tendance à utiliser la prière, « la nature » pour contrer cette attaque.

**Conclusion:**

une approche multidimensionnelle est capable d´offrir des voies de « libération ». Aussi, l´étude soulève une fois de plus la place de la médecine traditionnelle dans les systèmes sanitaires et montre le lien étroit qui existe entre la médecine traditionnelle et la spiritualité.

## Abstract (en)

**Introduction:**

since its appearance, the COVID-19 has exhausted global health systems. It was predictable that countries with weak health systems will be severly wiped out by the pandemic. Countries across Europe faced severe human loses and it was foreseable that Africa will experience an even worse tragedy. Suprisingly, since the evolution of the pandemic, there has been remarkable resistance from African countries, including Cameroon.

**Method:**

the study was phenomenographic. The data were collected successively from media observations (in particular the WHO site, national TV (CRTV) programs 'Parlons COVID'), social networks - Facebook and Whatsapp) and direct observations of some quarters of Garoua (Roumdé-Adjia, Foulbéré, Kakataré) and Mora for the Far North and the southern zone of Yaoundé (Ngoa-Ekelé, Nkolondom, Mokolo). These observations were associated with individual interview, reviews and note-taking around places of public circulation (places of worship, markets and discussion sites (Faada). The theory of functionalism was mobilized in this study.

**Results:**

the results show that Cameroonians perceive the pandemic as an eminently metasocial phenomenon which explains their tendency to use prayers, nature to counter this attack.

**Conclusion:**

the study suggests that a multidimensional approach is capable of offering avenues of « liberation ». Also, the study once again raises the place of traditional medecine in health systems and shows the close link that exists between traditional medicine and spirituality.

## Introduction

Dans une publication sur les réseaux sociaux (Facebook et Whatsapp) on peut lire une publication attribuée à l´Organisation Mondiale de la Santé (OMS): « L´OMS cherche à comprendre la surprenante résistance de l´Afrique à la pandémie de la COVID-19 ». Au bas de cette publication, et en guise de réponse, un « Africain » répond: « L´Afrique cherche à comprendre pourquoi l´OMS cherche à comprendre la surprenante résistance de l´Afrique face à la pandémie de la COVID-19 ». Cette question, d´une affligeante banalité, dans l´environnement des médias sociaux comme Facebook, atteste, en réalité, d´une profonde préoccupation scientifique à la fois des sciences humaines et sociales de la santé, mais aussi des sciences de la nature, à mesure que le coronavirus avance. Elle réinterroge, à de nouveaux frais, les problématiques sur la science et la religion, les postures science-Dieu, pratiques religieuses et connaissances scientifiques [1]. Selon les prévisions de l´OMS au début de la pandémie, les pays d´Afrique devaient enregistrer plus de décès que les sociétés de l´Europe Occidentale et de l´Amérique du Nord. A la grande surprise générale, à la grande société de mort, la société Africaine, dans son ensemble, les villes, les campagnes et les brousses du Cameroun en particulier ont opposé une réponse de « refus de mort ». Au 10 octobre 2020, selon les chiffres du ministère de la santé publique Camerounais, 194 pays au monde étaient touchés, soit 35 958 084 cas confirmés, avec 1 051 974 décès et 3% de taux de létalité. Pour ce qui est de l´Afrique en général, 47 pays sont concernés, soit 1 527 003 cas confirmés avec 36 805 décès et 2,4% de taux de létalité. Au Cameroun, il y a 21 203 cas confirmés avec 423 décès, 21 117 personnes guéries (95%), taux de létalité 2% et taux de sévérité 0,4% ont été enregistrés. Ces faits chiffrés sont des indicateurs probants de l´ampleur de la pandémie à l´échelle mondiale.

Le continent Africain, habituellement dénoté le continent le plus pauvre semble « mieux gérer » la crise sanitaire actuelle, on pourrait même parler du « miracle Africain ». Ce constat suscite plusieurs interrogations à la fois de surprise et de curiosité parce d´après l´OMS le coronavirus est 20 fois moins mortel en Afrique qu´en Europe [2]. Dans la littérature plusieurs thèses circulent sur la résistance de l´Afrique au corona virus. Une hypothèse, un peu plus ancienne, inspirée par les travaux de Didier Raoult, est que la présence du paludisme et la prise des antipaludéens par les Africains ont développé chez ces derniers des gènes, des anticorps capables de neutraliser le coronavirus. D´autres travaux insistent sur la circulation d´autres formes de coronavirus pendant les grippes saisonnières de mars, octobre et juillet. Cela sous-entend que des coronas virus différentes de la COVID-19 circulent déjà chaque année dans les pays africains, ce qui aurait amené à la résistance spectaculaire des Africains. L´OMS avance d´ailleurs plusieurs hypothèses. Une de ces hypothèses est liée à l´âge et au mode de vie des Africains: 91% de la population Africaine est jeune, elle a moins de 60 ans et l´âge médian est de 20 contre 30 pour le reste du monde; et les vieillards représentent moins de 5% des Africains. Le mode de vie rural, les habitats et espaces ouverts, et le voyage réduit à cause de la pauvreté peuvent aider à comprendre la résistance de l´Afrique, au sens de l´OMS (2020), sans oublier la non-existence des maisons de retraite (EPHAD) qui sont finalement des lieux clos où le coronavirus contamine plus rapidement. Une autre réflexion de l´OMS va dans le sens des gênes préhistoriques: les Africains n´ayant plus les gênes aliénés par la migration de l´espèce humaine de l´Afrique vers les autres continents. Toutes ces observations et bien d´autres ne sont pas comprises et entendues comme telles par les campagnards des pays Africains au sud du Sahara et plus précisément par les camerounais. Des réalités de la résistance et un vocabulaire de la COVID-19 sont construits et bricolés par les gens du bas. Ce sont ces construits sociaux, ces bricolages du monde d´en bas au quotidien de la résistance du Cameroun face à la COVID-19 que nous cherchons à intelligibiliser dans cet article. Comment donc comprendre la résistance du Cameroun à la COVID-19 en partant du point de vue des populations concernées elles-mêmes? Peut-on avoir mieux que la phénoménographie pour comprendre cette résistance?

## Méthodes

L´étude est une restitution phénoménographique de la COVID-19 dans les villes de Yaoundé, Garoua et Maroua et leurs environs entre les mois de décembre 2019 et d´octobre 2020. Les données ont été collectées de façon successive, des observations de médias (notamment les sites de l´OMS, télévisions Crtv (l´émission Parlons COVID), les réseaux sociaux (Facebook et Whatsapp), des observations directes de quelques quartiers de Garoua (Roumdé-Adjia, Foulbéré, Kakataré), Mora pour l´Extrême-Nord, et la zone Sud de Yaoundé (Ngoa-Ekelé, Nkolondom, Mokolo), associés aux entretiens individuels, recensions et prises de notes aux abords des lieux de circulation publique (lieux de culte, marchés et les espaces de causerie; les Faada) des personnes qui n´étaient pas à priori concernées par l´étude dans la mesure où « l´acte phénoménographique est donc directement associé à une focalisation sur des individus, pris séparément. Ce sont des individus qui intéressent d´abord la phénoménographie, non des actions, non des expériences: ce sont des individus qui existent, dans la succession des instants (les séquences de vie) et qui ainsi sont toujours plus que telle action, telle interaction et telle expérience » [3]. Cette posture a notamment permis de recenser ce que tel ou tel individu pouvait penser de la « résistance de l´Afrique » à la COVID-19, dans les moyens de transport en commun (taxi de ville, bus, etc.), aux abords d´un terrain de football, lors d´un jogging du dimanche, etc. Cette recherche s´inscrit dans le cadre théorique du fonctionnalisme. La théorie du fonctionnalisme postule qu´il existe une relation d´interdépendance entre les différentes composantes d´un système [4]. Elle sert à comprendre comment la société parvient à maintenir sa cohésion. En transposant cette théorie à notre étude, nous avons voulu saisir comment les différentes anthropotechniques ont aidé les camerounais à “survivre” face à la menace de la pandémie.

A Yaoundé, les observations-écoutes, prises de notes et recensions des actes phénoménographiques ont été faites au marché de Mokolo, le petit stade de Biyem-Assi, les taxis en commun circulant entre les quartiers Ngoa-Ekelé et Tam-Tam weekend, le bus de transport en commun de la ligne N°3 quittant la Poste Centrale pour la ville de Soa, et le trajet de jogging du dimanche: Palais de congrès-Mbankomo. A Garoua, elles ont été effectuées à l´hôpital régional (le service d´hémodialyse et le café d´en face), la *Faada* de Yérima (lieu de rencontre des enseignants et autres éducateurs), au quartier Bibemeri en face du lycée classique et moderne de Garoua et dans les cabarets (Périphérique Bar, Bar Collabo). A Maroua, les informations ont été collectées en sillonnant la rue kakataré (de Mont-Mandara Voyage au Service du Gouverneur) et à Mora (le quartier Igagoua (pont) a été mis à profit. L´entretien semi-directif a aussi été mis à profit. Cet entretien donne la possibilité à l´interviewé de s´exprimer autant que possible sur le thème de l´entretien. Cela nous a permis d´obtenir autant de verbatim possible et de faire une analyse de ce à quoi les camerounais attribuent leur résistance à la COVID-19.

Pour un souci de triangulation des informations obtenues par l´observation-écoute, 24 entretiens semi-structurés supplémentaires ont été conduits suivant un échantillonnage de milieu dont 4 avec des jeunes pousseurs de Garoua, 8 avec les étudiants de l´université de Yaoundé I, 4 personnels soignants de l´hôpital régional de Garoua (que nous avons interviewé sans leur signaler que nous étions en train de collecter des données), 4 vendeuses à la sauvette aux abords du marché Mokolo et 4 enseignants dont 2 des écoles normales et 2 de l´école primaire. En plus de ces entretiens semi-structurés, une séance d´entretien libre a été mise à contribution avec 24 élèves de la classe baccalauréat de l´Enieg bilingue de Garoua (session 2020-2021). Nous avons profité de notre position d´enseignant pour « ouvrir un débat » sur la relative épargne du Cameroun par le coronavirus. Les réponses des étudiants, à tour de rôle, ont été consignées et associées aux entretiens précédemment administrés. Les données collectées ont été analysées suivant les techniques d´analyse paradoxale et référentielle. Le passage des experts sur certains médias sur les chaines de télévision dans les débats du dimanche sur la thématique de la COVID et les émissions dédiées sont aussi mis à profit et codés (tel#) et tous les autres entretiens apparaissent sous le code (cov#) dans ce papier.

## Résultats

**Le Dieu des pauvres, un acteur invétéré du miracle Camerounais face à la COVID-19:** le dépouillement des données collectées lors des observations des cabarets, des discussions fortuites glanées ici et là dans les lieux de socialisation au Cameroun, sur les réseaux sociaux et même dans certains médias plus anciens, et des entretiens ont permis de faire émerger un thème principal sur « le retour du religieux dans sa grandeur et sa majesté ». Les camerounais demeurent fermement attachés à l´idée de ce que « les voix du seigneur sont impénétrables ». La plupart des propos soutiennent que si « la COVID-19 ne ravage pas les gens en Afrique c´est le fait divin » (Cov#4). Et que d´ailleurs, « Dieu qui est miséricordieux a compris les pleurs de ces enfants, qui pour la plupart sont dans le dénuement total et il ne pouvait pas leur ajouter la souffrance de la COVID-19 » (Cov#3). « Dieu est sage, il n´est pas fou. Tu crois qu´il peut ajouter la mort sur la souffrance? » (Cov#19). Les nosologies situent aussi la résistance dans la main invisible du Dieu, le Dieu de l´Exode est capable d´épargner les souffrances au peuple respectueux de ses principes. Si le Cameroun est épargné, alors que l´Organisation Mondiale de la Santé [baptisé pour l´occasion Organisation Mondiale de la Sorcellerie]) avait prévu « une déroute mortelle pour des pays aux structures hospitalières inexistantes, aux personnels soignants mal formés et outillés » (cov#13). C´est que les Africains sont sous le manteau de la constante providence divine. Cette croyance se renforce davantage dans l´esprit de ces derniers lorsqu´ils constatent que la majorité des hôpitaux n´avait pas un plateau technique assez large pouvant leur permettre d´accueillir des patients en besoin d´assistance respiratoire.

La résistance à « l´attaque » de la COVID 19 est une récompense divine. Cette récompense se mesure du point de vue social par une vitalité de la religiosité des camerounais de tout bord et de toutes les couches sociales. « Quand nous on fait nos fire fire [feu, feu] ici là, les gens croient que nous sommes fous »; « Dieu est avec nous, il n´a jamais quitté l´Afrique, moi je sais qu´en fait, il a djoss [dit: traduit du camfranglais] que comme vous êtes wise [sage: camfranglais] prenez aussi votre signe de respect mes mounas [enfants] » (cov#12). De toute façon, « Si les pays Africains résistent c´est parce que les Africains sont aimés et aiment leur créateur, nous on ne fait pas les mauvaises choses, on prie, chaque jour. Partout où tu passes, il y a au moins une église, une mosquée, est-ce que tu as vu un jour le tsunami au Cameroun? Dieu est avec nous! » (Cov#3). « Il va toujours nous délivrer du mal » (cov#24).

Dans une sortie médiatique, un gouverneur du Cameroun qui était infecté au virus a eu les mots suivants: « C´est Dieu, c´est Dieu qui est fort, qu´il soit loué, c´est lui seul qui m´a sorti de cette situation » (tel#2). L´appel exprès à Dieu est, d´ailleurs, fait. Certaines villes septentrionales comme Garoua, Maroua, qui ont vu d´un mauvais œil, la fermeture des lieux de culte (l´OMS a préconisé les règles de distanciation physique, les mosquées et autres églises qui sont des lieux touffus devaient donc être fermés) ont organisé, souvent une fois par semaine, des séances d´intercession pour que ces villes soient épargnées. La fermeture d´un lieu de culte, de façon provisoire, dans le Mayo-Louti (Nord Cameroun) a entrainé des émeutes. Cela prouve combien, bien que cela soit déjà su, une « sorte d´incrimination-responsabilité de Dieu » dans la résolution de la crise du COVID. Le Dieu qui les a maintenus dans la pauvreté, la sècheresse, et la violence de l´Etat ne pouvait pas, selon les interprétations des jeunes émeutiers, permettre que le même Etat insouciant les dépossède de tout ce qui leur reste: Dieu. Une construction très répandue de la survenue du « virus incompris » est rattachée à l´idée de la colère divine. La COVID-19 qui est une « flèche divine recherchant les mécréants » ne pouvait donc en aucun cas, frôler les « pieux », eux dont le quotidien rime avec louange divine et problèmes de survie. Cette pandémie, « Brutale et subite, elle seule, sait d´où est-ce qu´elle sort et où elle va. Mais Dieu seul est notre secours et garant contre cette chose » (tel#3). « Qu´est ce qui peut nous aider si ce n´est Dieu »? « Cette maladie qui est née en Chine fait ses études en Europe, travaille aux USA, va mourir s´il plait à Dieu en Afrique » (cov#6), « il ne fallait pas qu´il vienne en Afrique, il sera vaincu au nom de Jésus » (cov#21).

**Les sorciers Camerounais ont-ils servi, pour une fois, à faire du bien?** Au début de la survenue de la COVID-19, suite à sa qualification en pandémie autour du mois de mars et de la désolation et, même la peur du lendemain dans laquelle elle inscrit l´humanité toute entière, certains « intellectuels africains » et d´autres acteurs de la société civile avaient estimé que pour « Une fois, il faut que les marabouts, les sorciers et autres tradipraticiens africains servent à quelque chose » (tel#4). Dans les faits, à mesure de l´évolution de la pandémie, des solutions miracles des plus fantaisistes aux recettes magiques tues et occultées ont prospérées. Des sorciers ont émergé et tapé fort la poitrine sur « l´efficacité certaine, mais indémontrable de leurs produits ». Ce méli-mélo, ce fourre-tout thérapeutique, que crée le chaos pressant d´une éventualité agressive d´un coronavirus dévastatrice, a induit la construction d´une puissance sorcière Africaine capable d´éliminer cette dernière dans la vie des gens du bas. Cela s´est manifestée notamment par un retour forcé des camerounais de la diaspora (européenne) vers les terres camerounaises et un repli tactique de ceux installés dans les villes vers les villages; terres des ancêtres, mais aussi terres par excellence des prouesses des sorciers (personnes dotées des pouvoirs surnaturels capables de miracles, Dieu, Maitre de sa propre science, capable de la mettre au service du bien comme du mal). Une idée généralement répandue dans les constructions sociales de la sorcellerie est que: ceux qui sont dépositaires de ce savoir mystique sont, pour la plupart, faiseurs du mal, que ce sont eux qui sont à l´origine des « Blocages des enfants des gens », « lancent les malchances aux gens » (tel#2), « Mangent les gens et sucent le sang des gens, enlèvent le sexe des gens et rendent stériles » (cov#6), laquo; C´est même connu qu´ils peuvent empêcher que la pluie tombe dans un village » (cov#21).

L´idée de la sorcellerie au Cameroun est associée aux pratiques de méchanceté, de mal et de danger; c´est ce qui est répandue dans la société. Les sorciers sont donc des méchants, des personnes dangereuses et c´est ce que connaissent les populations. De façon unanime, les populations, face au péril général COVID-19, « ont exigé que les sorciers qui avaient passé tout leur temps à faire du mal, devaient se recycler pour faire du bien au nom de la préservation humaine » (cov#2 et tel#2). « A quoi nous servent ces sorciers, s´ils ne sont pas capables de freiner la propagation du COVID-19? » (cov#23). « Ils ont passé tout leur temps à tuer les gens et nous ont bloqué, maintenant qu´ils montrent aux gens qu´ils sont forts, sinon nous allons tous mourir » (cov#13). Autant, il est difficile de prouver, à partir des imaginaires populaires le rôle joué par les sorciers africains dans la résistance au COVID-19, autant l´idée, quoi que incohérente, comme la sorcellerie d´ailleurs, d´une main invisible dans le développement des cas de COVID-19 (l´état très bas des patients infectés, leur décès) demeure très présente auprès des camerounais.

**Le Cameroun: terre des miracles:** les participants à l´étude demeurent fermement attachés à l´idée de ce que le continent africain et le Cameroun est la terre des impossibles, la terre de l´extraordinaire. C´est donc une situation qu´il ne faut pas chercher à expliquer. Même ceux qui cherchent à « creuser scientifiquement » la question de la progression de la pandémie à coronavirus lancent « scientifiquement ça ne s´explique pas » (tel#5). « Il y a des thèses nombreuses et différentes qui circulent, mais aucune n´est scientifiquement plausible » (tel#6). Le fait que le Cameroun soit épargné par la COVID-19 n´est donc que « pur miracle » et que de toute façon « c´est Dieu qui fait ses choses mon frère jusqu´à, un temps, ils ont dit que quand l´histoire-là [corona] va arriver ici nous allons mourir comme des cafards. C´était mal connaitre Dieu » (cov#15). L´idée du miracle camerounais trouve aussi sa sagacité chez les gens du bas du fait des prévisions, des décisions, parfois contradictoires, des instances mondiales de la santé en ce qui concerne, par exemple, la distanciation physique, le port de masque et l´utilisation des stérilisants hydroalcooliques. L´OMS qui hésitait en ce qui concerne l´efficacité réelle de ces trois postures dans la prévention réussie de la pandémie.

**Le Cameroun n´a pas encore dit toute sa science:** la médecine traditionnelle Africaine, bien que peu sophistiquée comparativement à d´autres médecines traditionnelles (l´on notera sans prétention de classification, par exemple, que la médecine traditionnelle japonaise est enseignée dans les grandes écoles) est, au sens des participants, pour quelque chose dans la résistance des gens au COVID-19. Grace à certaines coctions et autres portions, certaines personnes affirment défier le coronavirus sur ses terres de prédilection. « Je suis allé en Italie, en France, le corona ne m´a rien fait [en brandissant une bouteille de recueils d´écorces acres et amers: *mfol*], le corona ne peut rien contre ça » (tel#8). Il y a, dans les plantes et les feuilles poussant sur les terres africaines, thérapie. « Moi, par exemple, je consomme de l´ail, du *djidja [gingembre]* et du citron, tantine coro [pour coronavirus] ne peut rien contre ces gens [plantes et fruits suscités] » (cov#18). Face au corona virus, même les « soignants » de la médecine moderne se tournent vers « le traditionnel ». Une infirmière, ayant survécu au corona virus qui était en isolement à Bocklé (périphérie Sud de Garoua), soutient d´ailleurs « qu´on nous donnait même quoi là-bas, c´est juste que le matin, à midi et le soir il fallait boire son djidja. C´est ça qui nous a sauvé » cov#14.

Les arbres, feuilles, écorces et autres herbes amers ont été mis à contribution. Des images mettant en exergue ces thérapies sont commentées sur les réseaux sociaux comme les prouesses inexplicables de cette « médecine Africaine» qui défie tout pronostic.

**Dieu est aussi dans la chloroquine:** des contenus des discours des « prophètes des églises de réveil » captés sur certains médias, « des discussions des quartiesards », des « débats dominicaux des experts sur les chaines de radio et télévisions camerounaises », « des discussions des femmes bayamsellam » des marchés de Mokolo, une idée de la prise de position de Dieu en faveur des enfants de l´Afrique se dessine. L´idée servant de talon d´Achille à cette réalité est que « pendant longtemps, les firmes occidentales ont imposé à l´Afrique le paludisme et, en retour, revendent les médicaments aux africains ». Le marché de la chloroquine qui était prévu pour nuire aux africains a le dessein, aujourd´hui, de les soigner. L´on affirme dès lors qu´: « ils ont développé le marché du paludisme pour nous tuer, aujourd´hui Dieu nous dit que c´est ça qui va nous soigner ».

## Discussion

**Le Dieu des pauvres, une construction socio-anthropologique par le recours à l´imaginaire collectif:** l´anthropologie sociale offre un cadre théorique fécond à la compréhension des pandémies et des maladies infectieuses de façon générale [5]. Elle révèle « la façon dont des faits morbides sont imbriqués dans de plus vastes systèmes sociaux et comment du plus simple au plus complexe, la récurrence de pratiques apparemment modestes configure de larges paysages sanitaires » [6]. Ce regard éloigné permet de souligner de fortes corrélations entre les caractéristiques des contextes culturels, sociaux, économiques et les prévalences des pathologies infectieuses. Les causes constituantes de la maladie ne se limitent donc pas à une définition médicale. Pour autant que: *« Dans l´imaginaire social, le diagnostic biomédical peut être banal, la cause vraie pouvant être extérieure à l´organisme malade »* [7]. C´est pourquoi il importe-complémentairement aux données « dures » des sciences fondamentales et à d´indispensables propositions thérapeutiques-de rendre compte des pathologies du point de vue du « tout social » et surtout, de tirer les conséquences opérationnelles de cette perspective anthropologique [6]. Dans la mesure où le monde d´en bas en matière de (ré) composition de l´espace public ne manque pas d´initiatives [8]. En effet, la pandémie du COVID-19 a entrainé un doute dans l´analyse des enjeux sociaux qui tendent à être rapidement comblé par les acteurs traditionnels comme les pouvoirs publics (L´Etat du Cameroun, l´OMS) qui se trouvent dans l´incapacité de prouver les causes réelles de cette résistance autres que les « hypothèses ». Ce qui conduit à un braconnage de la compréhension de cette résistance par le recours à l´imaginaire collectif.

Dieu n´est donc pas une fatalité, mais bien une construction de la réalité de la résistance à la pandémie du COVID-19 dans les villes camerounaises. C´est une réponse bricolée, un métarécit porté par ses adeptes, forcé souvent par ses promoteurs à des communautés parfois en manque de « vérité » sur la réalité de cette pandémie. Puisqu´effectivement: qu´est-ce la COVID-19, la réponse est généralement: « personne ne sait », mais chaque jour elle crée de nouvelles surprises. L´idée de Dieu tire aussi sa construction dans une charge de revanche vis-à-vis des plus forts, notamment la figure de l´Etat, les organismes internationaux (OMS) qui ont pendant longtemps maintenu, au sens des populations, des diktats qui arrangeaient peu leurs situations de vie. L´érection de Dieu dans la résistance de l´Afrique au COVID est un souhait collectif de se retrouver, pour une fois, du côté des plus forts (Dieu) pour narguer les plus faibles (l´Etat insouciant et l´OMS qui est suspectée de faire le jeu des firmes pharmaceutiques), mais qui, par la force de Dieu justement, sont devenus faibles et incapables de freiner la propagation de la maladie, maladie qui paradoxalement ne touche que les plus riches; « ceux qui peuvent partir chez les blancs, prendre l´avion », « qui sont loin de souffrances quotidiennes ». Dans la construction de la vérité sociale, « les gens de moins de rien » [9] n´hésitent pas à construire Dieu, soit pour le mettre face à une responsabilité, qui dans les représentations sociales, ne peut être valablement accomplie que par lui, donc, « l´incriminer », soit pour chercher à travers lui, les possibilités d´un dénouement dans la société du risque dans laquelle se trouvent ces personnes; dans ce cas, un salut, une issue heureuse est recherchée. Un peu comme dans le cadre des camerounais dans l´interprétation de la résistance au COVID, il a été observé dans certaines circonstances « l´incrimination » et « le rôle de Dieu » dans une situation de pauvreté où l´eau, le mil et la terre deviennent des questions de vie ou de mort [9].

**Dieu et la pandémie de la COVID-19:** les résultats de la présente réflexion ont permis de ressortir que l´explication la plus plausible de la relative épargne des villes camerounaises est l´œuvre de Dieu. Les réponses obtenus montrent que pour les camerounais c´est Dieu, pas la science, encore moins l´OMS et ses stratégies ni moins « l´Etat insouciant du Cameroun » qui a aidé à ce que « les camerounais ne meurent pas en nombre important ». Ces résultats confirment ceux trouvés par des sociologues américains [10,11]. D´après ces auteurs, aux Etats Unis d´Amérique, les populations les plus religieuses étaient spécialement enclines à ne pas respecter les recommandations de lutte contre la pandémie parce qu´elles avaient une vision négative de la science et des scientifiques et une grande considération pour les croyances religieuses concernant la pandémie elle-même. Les auteurs ont montré que, pendant la pandémie, les croyances religieuses ont servi de fond de scène idéologique (nationalisme chrétien), aux populations pour résister aux recommandations médicales. Certains pasteurs ont d´ailleurs fait croire que la COVID (Christ Over Viruses and Infectious Disease) [12] est liée à la « désobéissance nationale aux lois de Dieu », et « Que Dieu pourra apporter la paix si la société se repend des maux qui minent la société ». Le coronavirus est donc, au sens de ces populations, une punition divine contre la société américaine contre son comportement immoral. Pour mettre en exergue le rôle de Dieu dans la pandémie, les sociologues ont expliqué que les américains de droite étaient ceux qui respectaient le moins les prescriptions médicales et avaient tendance à sortir, prendre le bain de foule et fouler toutes les recommandations des scientifiques au pied. Ceux de gauche étaient plus respectueux des recommandations scientifiques. En Chine [1], le discours répandu est que: « l´épidémie est un démon », et pour le terrasser, il fallait un Dieu; Dieu lui-même devait s´occuper de cette pandémie. C´est pour cela que les hôpitaux construits par les autorités chinoises portent le nom de Fire God Mountain Hospital et Thunder God Mountain Hospital.

**Limites de l´étude:** notre étude s´est déroulée dans trois régions du Cameroun sur les dix. On aurait certainement mieux saisi les dynamiques internes et externes de cette socio-anthropologie de la résistance à la COVID si toutes les régions avaient été incluses dans l´étude. A cet effet, cette étude peut être prolongée pour mener une étude similaire d´envergure beaucoup plus grande dans toutes les régions. Au terme de ce travail, nous sommes loin d´éprouver le sentiment de satisfaction totale car nous reconnaissons que quelques aspects de notre recherche ont été occultées et il est souhaitable qu´il fasse l´objet des investigations futures.

## Conclusion

Le présent article rendait compte des différentes stratégies que mettent sur pied les camerounais pour « contenir » le COVID-19. Les différentes observations révèlent que les camerounais ont une marge de manœuvre qu´ils négocient au quotidien pour faire face à l´épidémie. Les invocations diverses dans les lieux de cultes, les concoctions, sont autant de manœuvre employés par ces derniers pour survivre à la COVID-19. Pour les Camerounais, la tragédie (maladie) relève d´une violation de la « nature» et doit être traitée comme un fait essentiellement culturel. Aussi au terme de ce travail il ressort que la médecine plurielle (traitement traditionnel, la prière) est importante dans l´amélioration du bien-être global de la personne.

### Etat des connaissances sur le sujet

- La COVID-19 est une crise sanitaire mondiale;
- La COVID-19 a causé d´importantes pertes humaines au niveau mondial.

### Contribution de notre étude à la connaissance

- Cette étude aborde de nouveau le débat sur les « médecines » en relevant que le bien-être n´est pas que « curatif » mais aussi spirituel et social;
- Un accent particulier pourrait être porté sur les espèces exploitées par la pharmacopée traditionnelle en ce moment précis de pandémie COVID-19 pour une meilleure gestion de la pandémie.

## Remerciements

Les auteurs tiennent à remercier sincèrement toutes les personnes qui ont donné leur avis favorable à partager avec eux des informations qui leur ont permis d´atteindre les objectifs de cette étude. Ils adressent aussi des vifs remerciements aux élèves de la classe baccalauréat de l´Enieg bilingue de Garoua (session 2020-2021).

## References

[1] Bretelle-Establet F (2020). la santé en Chine du Sud (1898-1928). CNRS Editions.

[2] WHO/AFRO (2020). COVID-19 situation external report 10. WHO.

[3] Piette A (2016). Anthropologie existentiale et phénoménographie: observer l´homme en tant qu´il existe. Anthropologie et société.

[4] Ritzer G (2008). Sociological theory. McGraw-Hill.

[5] Revel J, Jeux d'échelles (1996). La micro-analyse à l'expérience. Paris, Gallimard et Le Seuil, coll. Hautes Études.

[6] Jaffre Y (2006). Dynamiques et limites socio-anthropologiques des stratégies de prévention et de contrôle des risques infectieux dans les pays en voie de développement in La maitrise des maladies infectieuses, un défi de santé publique, une gageure médico-scientifique.

[7] Djouda Feudjio YB (2010). Réseaux relationnels et processus de soutien aux malades de la tuberculose au Cameroun. Revista hispana para el analisis de redes sociales.

[8] Abe C (2011). L´espace public au ras du sol en postcolonie: travail de l´imagination et interpellation du politique au Cameroun. Afrique et développement.

[9] Ela JM (1982). L'Afrique des villages, Karthala; Les Afriques edition.

[10] Hill TD, Kelsey Gonzalez, Amy M Burdette (2020). The blood of christ compels them: state religiosity and state population mobility during the coronavirus (COVID-19) pandemic. Journal of religion and health.

[11] Perry SL, Joshua B, Grubbs Andrew L, Whitehead (2020). Culture wars and COVID-19 conduct: christian nationalism, religiosity and Americans´ behavior during the coronavirus pandemic. Journal for the scientific study of religion.

[12] Curtis E (2020). Pastors corner: COVID-19-Joshua 1: 9. Havre Daily News 3/20.

